# Alcohol's involvement in an array of harms to intimate partners

**DOI:** 10.1111/dar.12435

**Published:** 2016-06-11

**Authors:** Anne‐Marie Laslett, Heng Jiang, Robin Room

**Affiliations:** ^1^ National Drug Research Institute Curtin University Melbourne Australia; ^2^ Centre for Alcohol Policy Research La Trobe University Melbourne Australia; ^3^ School of Population and Global Health University of Melbourne Melbourne Australia; ^4^ Centre for Social Research on Alcohol and Drugs (SORAD) Stockholm University Stockholm Sweden

**Keywords:** alcohol, intimate partner harm, harm to others

## Abstract

**Introduction:**

Harms from intimate partners' (IP) drinking range from frustration because the partner has not performed their role to assault.

**Aim:**

To describe the prevalence and persistence of alcohol‐related harms to IPs and assess which respondents are more likely to report discontinuation of this harm.

**Design and methods:**

Cross‐sectional (*n* = 2649) and follow‐up (*n* = 1106) alcohol's harm to others telephone surveys in 2008 and 2011 (response rates of 35% and 15% of the original sample respectively) were used to elicit harms to respondents from their IP's drinking (by gender and relationship). To examine discontinuation, a sub‐sample of 83 respondents was analysed in detail.

**Results:**

A total of 6.7% of Australians were negatively affected by an IP's drinking in 2008. Women were more likely to report harm than men from an IP's drinking. Of the 1106 respondents who completed both surveys, the majority (90%) reported no harm from IPs although 3% reported harm in both surveys. No significant correlates of discontinuation of harm were identified.

**Discussion:**

Many Australian relationships are affected in a range of ways because of the drinking of their IPs. A minority of respondents were affected by their IP's drinking, yet over half (57%) of those harmed in 2008 continued to experience harm in 2011. Additionally, half (46.9%) of those who were not harmed in 2008 but did live with a heavy drinking IP did go on to be harmed in 2011. More research on the role of alcohol‐related harm from IPs with larger samples is required to examine predictors of change. [Laslett A‐M, Jiang H, Room R. Alcohol's involvement in an array of harms to intimate partners. *Drug Alcohol Rev* 2017;36:72–79]

## Introduction

The effects of heavy drinking upon intimate partners (IP)—including partners, ex‐partners or boyfriends/girlfriends—extend to poor performance of one's role, ignoring partners' needs, disregard for their feelings, serious arguments, verbal abuse, and physical and sexual harm. Heavy drinking also causes health, financial and relationship problems for the drinker that extend to create substantial negative ripple effects for their IPs 1, 2, 3, 4, including substantial tensions between couples, arguments, divorce and domestic violence 5.

This study aims to measure the baseline prevalence in Australia of alcohol‐related harms to IPs, including violence, and further describe the experience of respondents affected by a partner's drinking, at a later time point.

Alcohol is involved in a substantial proportion of cases of harm to IPs both in and outside the household across many countries 6, 7. Leonard 8 estimated that 25–50% of IP violence reported in population surveys from across the USA involved alcohol. In Australia, around one in three (35%) recent IP violence incidents were alcohol‐related, with 32% of women reporting that their partner was drinking at the time of the most recent violent act 9. Drawing on the 2005 Australian Personal Safety Survey, it was estimated that alcohol contributed to 50% of all partner violence and 73% of physical assaults by a partner 10.

Research on IP violence in Australia has identified many of the difficulties female spouses living with heavy drinkers experience, including being verbally berated, intimidated, physically hurt, financially disadvantaged and isolated 11. Interviews with women in other countries who live with heavy‐drinking men also provide insights into the many problems they experience, including physical violence, verbal abuse and destruction of their belongings 5.

The role of alcohol in IP violence is still contested 12, and it is feared that describing alcohol as causal will remove responsibility from the drinking perpetrator 13. Alcohol is rarely a necessary or sufficient cause of violence, but on the other hand, the violence might not have occurred without the drinking and alcohol emerges as a consistent risk factor in its perpetration 14. Despite the lack of consensus about the aetiology of alcohol‐related IP violence, the association of heavy episodic drinking patterns with more aggression within relationships and increased severity of injury is consistent across several studies 15, 16, 17, 18. In a cross‐cultural study of 13 countries, Graham *et al.* 19 showed that women experience a heightened risk of partner violence on days that men have been drinking. Current policy responses argue for interventions that address both alcohol misuse and attitudes that are supportive of violence against women 20, 21.

Less is known about other common relationship problems linked to alcohol that affect female and male IPs in the general population 10. Moreover, few surveys have examined whether IP harms from others' drinking persist or recur, although findings from qualitative and treatment samples suggest many family members, and particularly spouses, often suffer over long periods before taking action or seeking interventions. To understand more about these alcohol‐related harms from IPs, the following research questions were asked:

1. What is the baseline prevalence of alcohol‐related harms to IPs, including violence?
2. Does this prevalence vary by gender and type of intimate relationship?
3. What percentage of respondents continued to experience harm from an IP's drinking in a follow‐up survey?
4. Among those negatively affected by a partner's drinking at baseline, which groups of respondents were more likely to no longer experience this harm in 2011?

## Methods

### Data

Cross‐sectional and follow‐up alcohol's harm to others surveys undertaken in 2008 and 2011 were used to track harms from others' drinking. In 2008, 2649 Australians aged 18 years or older were telephone interviewed. The sample was based on a national random sample generally representative of the national population of Australians aged 18 years or older 10. The 2008 survey response rate was 35% using strict American Association for Public Opinion Research guidelines and although poor, is commonplace across Australia. Young men and less formally educated Australians were slightly under‐represented, but this was corrected by post‐weighting in the 2008 sample. In 2011, the sampling frame of the follow‐up study is by definition limited to those participants who agreed to be re‐contacted in 2011 (*n* = 2304), and 1106 respondents were finally interviewed.

Thus, the response rate for the follow‐up survey was 42% (or 15% of the original sample). The study received ethics approval from Eastern Health Research and Ethics Committee in 2008 and 2011.

### Outcome and predictor variables

In both surveys, IPs were defined as including spouses, partners, ex‐partners and boyfriends/girlfriends. The primary harm outcome variable used in Table 1 was whether ‘the[ir] IP's drinking had negatively affected [them] in some way in the last 12 months?’. In Table 2, discontinuation of harm (harm in 2008 but not 2011) versus continuation (harm in 2008 and 2011) was the outcome variable. Respondents were next asked to nominate the person who had most negatively affected (or harmed) them. This subsample of respondents who reported their IP had most negatively affected them (analysed further in Table 3) was asked whether this person had adversely affected them a lot or a little, and additionally about the number of times they had experienced specific harms that included minor and more serious harms such as physical violence, verbal abuse, breaking or damage of something that mattered, forced sex and being put at risk in the car when their IP was drink‐driving (see tabulated results for the exact wording of these items). Responses were dichotomised into yes, affected one or more times, and no—not affected. In 25 cases where the IP's drinking was not the drinking that had most affected the respondent in 2011, respondents were not asked whether they had been affected a little or a lot, or about the specific harms. Consequently, these cases were not included in Table 3 and analyses assessing severity, and there are small discrepancies in earlier and later table numbers. Hence, in Table 3, the primary outcome variable was whether the respondent experienced continuation of harm in 2011 from an IP's heavy drinking or not, given their experience of each of the specific harms in 2008 (among the group of respondents who reported they were most negatively harmed by their IP's drinking in 2008 and 2011). This analysis was also undertaken for respondents experiencing two or more specific harms.

**Table 1:** Percentage of respondents identifying heavy drinking intimate partners and reporting negative effects from their drinking (2008 HTO Survey) (*n* = 2649)

	a) Have a heavy drinking intimate partner			b) Negatively affected by a heavy drinking intimate partner's drinking			c) % of respondents with a heavy drinking intimate partner negatively affected by the heavy drinking of their intimate partner		
	Male, % [95% CIs]	Female, % [95% CIs]	Total, % [95% CIs]	Male, % [95% CIs]	Female, % [95% CIs]	Total, % [95% CIs]	Male, % [95% CIs]	Female, % [95% CIs]	Total, % [95% CIs]
(*n*)	(1089)	(1560)	(2649)	(1089)	(1560)	(2649)	(108)	(265)	(373)
Intimate partner									
Spouse/partner (*n* = 54/168)	5.4 [4.1–7.1]	11.8 [10.1–13.7]	8.7 [7.6–9.9]	2.7 [1.7–4.1]	6.5 [5.3–8.1]	4.6 [3.8–5.6]	49.2 [35.2–63.4]	55.4 [47.4–63.1]	53.5 [46.5–60.4]
Ex‐partner (*n* = 44/81)	3.8 [2.7–5.4]	4.5 [3.5–5.8]	4.2 [3.4–5.1]	1.1 [0.6–1.9]	1.7 [1.1–2.5]	1.4 [1.0–1.9]	28.1 [15.5–45.3]	36.6 [25.4–49.4]	32.8 [24.0–43.1]
Boyfriend/girlfriend (*n* = 10/16)	1.1 [0.5–2.4]	1.4 [0.8–2.5]	1.2 [0.8–2.0]	0.4 [0.2–0.9]	0.9 [0.4–1.9]	0.7 [0.4–1.2]	34.9 [11.5–68.8]	66.1 [35.2–87.4]	52.6 [29.6–74.5]
Total with heavy drinking intimate partners	(108) 10.3 [8.4–12.6]	(265) 17.7 [15.7–19.9]	(373) 14.1 [12.6–15.7]	(45) 4.1 [3.0–5.7]	(131) 9.1 [7.6–10.1]	(176) 6.7 [5.7–7.8]	(45) 39.9 [29.9–50.9]	(131) 51.4 [44.7–58.0]	(176) 47.2 [41.6–53.1]

Percentages in this table were calculated on the total sample of 2649 respondents for columns a and b.Boyfriend/girlfriend includes current (*n* = 20) and ex‐boy/girlfriend (*n* = 6). Figures in bold indicate a significant difference in proportion by gender, *P* <0.05 using stata's lincom test of proportions.

CI, confidence interval.

**Table 2:** Numbers (and %) of respondents reporting alcohol‐related harms from intimate partners members by sociodemographic characteristics (*n* = 83)

Variables	2008 only–––discontinuation %	Both years–––continuation %	Predicted discontinuation of harm bivariate OR
(*n*)	(36)	(47)	(83)
Gender of respondents			
Male	30.6 (17.5–47.7)	27.7 (16.6–42.4)	Ref
Female	69.4 (52.3–82.5)	72.3 (57.6–83.4)	0.9 (0.3–2.5)
Age 2008^a^			
18–35	13.9 (5.7–29.9)	8.5 (3.1–21.0)	Ref
36 and over	86.1 (70.1–94.3)	91.5 (79.0–96.9)	1.0 (0.4–2.5)
Neighbourhood affluence			
(4 missing)^b^			
Disadvantaged	62.9 (45.5–77.4)	48.9 (34.8–63.3)	Ref
Less disadvantaged	37.1 (22.6–54.5)	51.1 (36.7–65.2)	0.6 (0.2–1.3)
Respondent drinks 5+ at least monthly in the past year–––2008			
Yes	63.9 (46.8–78.1)	70.2 (55.4–81.7)	Ref
No	36.1 (21.9–53.2)	29.8 (18.3–44.6)	1.3 (0.5–3.4)
Respondent drinks 5+ at least monthly in the past year–––2011			
Yes	75.0 (58.0–86.7)	66.0 (51.0–78.3)	Ref
No	25 (13.3–42.0)	34.0 (21.7–48.9)	0.7 (0.3–1.7)

Test of proportions found no significant differences between groups within columns. Bivariate logistic regression results suggest there is no significant difference by gender, age, neighbourhood affluence and respondent drinking pattern (5+ standard drinks at least monthly) when predicting experience of discontinuation of harm from IPs.

a Age collapsed to two categories in this table because of small numbers.

b In this study, the Socio‐Economic Indexes for Areas of Disadvantage is used; it measures how disadvantaged an area is compared with other areas in Australia (ABS, 2006) and allocates a score for each postcode. Disadvantage is measured on a scale of 1 to 5, where 1 is the most disadvantaged, and 5 is the least disadvantaged. Here, the scale was recoded into two groups of roughly equal size, ‘Disadvantaged’ (score of 1–3) and ‘less disadvantaged’ (score of 4–5, used as the reference category).

IP, intimate partner; OR, odds ratio.

**Table 3:** Percentages of respondents most affected by intimate partners within discontinuation and continuation of harm groups experiencing specific harms from intimate partners and odds ratios for continuation of harm (*n* = 58)

So how many times in the last 12 months…	Per cent specifically harmed in 2008 only–––discontinuation	Per cent specifically harmed in both years, 2008 and 2011–––continuation	Prediction of continuation of harm bivariate OR
(*n*)	(24)	(34)	(58)
Did you have a serious argument that did not include physical violence because of their drinking?	66.7 (45.2–82.9)	76.5 (58.8–88.1)	1.6 (0.5–5.2)
Did you feel threatened because of their drinking?	29.2 (14.1–50.8)	26.5 (14.0–44.2)	0.9 (0.3–2.8)
Were you emotionally hurt or neglected because of their drinking?	58.3 (37.5–76.6)	79.4 (62.0–90.1)	2.8 (0.9–8.8)
Were you physically hurt because of their drinking?	12.5 (3.9–33.6)	5.9 (1.4–21.6)	0.4 (0.7–2.8)
Did you have to stop seeing them because of their drinking?	29.2 (14.1–50.8)	24.2 (12.3–42.2)	0.8 (0.2–2.5)
Were you put at risk in the car when they were driving because of their drinking?	0.0	6.1 (1.4–22.2)	‐
Were you forced or pressured into sex because of their drinking?	4.2 (0.5–26.0)	5.9 (1.4–21.6)	1.4 (0.1–16.8)
Did they negatively affect a social occasion you were at because of their drinking?	66.7 (45.2–82.9)	58.8 (41.3–74.4)	0.7 (0.2–2.1)
Did they fail to do something they were counted on to do because of their drinking?	45.8 (26.8–66.2)	56.3 (38.4–72.6)	1.5 (0.5–4.4)
Did they break or damage something that mattered to you because of their drinking?	13.0 (4.0–34.7)	12.1 (4.5–29.0)	0.9 (0.2–4.6)
Could you not bring friends home because of their drinking?	28.6 (10.3–58.1)	20.0 (8.2–41.3)	0.6 (0.1–2.9)
Did they not do their share of their work around the house because of their drinking?	42.9 (19.4–70.0)	34.6 (18.4–55.3)	0.7 (0.2–2.7)
Did you have to leave home or sleep somewhere else because of their drinking?	21.4 (6.5–51.7)	23.1 (10.3–43.9)	1.1 (0.2–5.3)
Was their less money for household expenses because of their drinking?	21.4 (6.5–51.7)	36.0 (19.2–57.1)	2.1 (0.5–9.4)
Two or more of the items earlier	83.3 (62.0–93.9)	88.2 (71.8–95.7)	1.5 (0.3–6.7)

Test of proportions found no significant differences between groups within rows. Bivariate logistic regression results suggest there is no significant difference by type of harm experienced in 2008 when predicting experience of continuation of harm from intimate partners.

OR, odds ratio.

Demographic information on the respondent, such as age, gender, neighbourhood affluence and employment status, was also collected. Neighbourhood affluence was measured using the Socio‐Economic Index for Areas for Disadvantage 22 based on the respondent's postcode. Because of small numbers, respondents were put into two groups of roughly equal size, high disadvantage with a score of 1 to 3 and low disadvantage with a score of 4 or 5. Alcohol consumption of the respondent, not the harmful drinker, was assessed using a response to the frequency of 5+ Australian Standard Drinks (10 g ethanol), which was converted to the number of occasions per year. Only demographic information from the 2008 survey was used.

### Weighting and statistical analysis

Our 2008 Survey estimates use sampling weights and post‐stratification weighting. However, in the follow‐up analyses of 2008 and 2011 survey data, unweighted data were used to predict whether harm to the respondent from an IP's drinking was present in 2011. Given the response rate of the 2011 survey, it was deemed inappropriate to apply sampling weights to these data. Table 1 results are both sample‐ and post‐weighted as they are sourced from the 2008 survey; however, in the results presented in Table 2 and 3, the grouping variable is based on the 2011 data, as such any respondents who did not participate in 2011 were not included, and sampling and post weights were no longer appropriate. All data analysis was conducted on stata version 14. stata's tests of proportion were used to test differences between two groups, that is, whether gender (of the respondent) was associated with having been negatively affected by an IP, and whether the group who experienced harm in both years was different from the group that experienced harm in 2008 and then again in 2011 (or not in 2011). Bivariate logistic regression analyses were also used to examine correlates of continuation (and discontinuation).

## Results

A total of 14% of the 2008 survey sample reported having a partner, ex‐partner or boyfriend/girlfriend who drank heavily (regularly or occasionally) in the last 12 months (Table 1). Women were more likely than men to report having a heavy drinking partner (this difference was only identified in the spouse/partner group). Table 1 also shows that as a percentage of the overall sample, 6.7% of respondents indicated that they were negatively affected (or harmed) by the drinking of an IP. Female respondents were statistically significantly more likely to report being negatively affected by an IP's drinking than male respondents (9.1% vs. 4.1%), although this was not the case for boyfriends/girlfriends (0.4% vs. 0.9%) or for ex‐partners (although numbers were small in these groups). Around 1.9% of the sample was negatively affected ‘a lot’ by the drinking of an IP, and again women were statistically significantly more likely to report being negatively affect a lot than men (3.4 vs. 0.4%—results not shown here). Of the respondents who reported having a heavy drinking IP, nearly half (47.2%) reported that they had been negatively affected by their heavy drinking IP's drinking in the past 12 months, with no statistically significant difference between the proportion affected in women (51.4%) and men (39.9%). Examining the estimates within columns, a higher percentage of respondents with a heavy drinking spouse reported they had been negatively affected by the drinking of their spouse in the past 12 months, compared with ex‐partners in both the overall group (4.6% vs. 1.4%) and the sub‐group where the denominator only included heavy drinkers (53.5% vs. 32.8%).

### Negative effects (harm) from intimate partners in the 2008 survey and the 2011 follow‐up survey

By dividing the 1106 respondents in 2011 based on their experience of harm from an IP in 2008 and 2011 four groups were defined:

- Not harmed by an IP in either year (*n* = 993, 90.4%);
- Harmed in 2011 but not in 2008 (*n* = 30, 2.9%);
- Harmed in 2008 but not in 2011 (*n* = 36, 3.7%)—discontinued harm;
- Harmed in both years (*n* = 47, 3.0%)—continued harm.

The focus of the remainder of analyses is comparing these last two groups—those who are no longer harmed (discontinued harm group) and those who are continuing to be harmed—to see if there is anything in their experience in 2008 that could predict their experience of harm in 2011.

As can be gathered from this, the majority of the sub‐sample (56.6%) harmed in 2008 reported that they were harmed by an IP in 2008 and again in 2011. The data also enabled us to answer the question, ‘Of those living with heavy drinking IPs who were not harmed in 2008 (*n* = 197), what proportion went on to be harmed in 2011?’. However, this was a small group, and only 64 completed the survey in 2011, with 30 of these reporting being harmed in 2011. In other words, almost half (46.9%) of those who were not harmed in 2008 but lived with a heavy drinker did go on to be harmed in 2011. This group was not analysed further because of small numbers.

Table 2 presents more detailed information on the sub‐sample of the 83 respondents who reported either that they had been harmed in 2008 and were no longer or, in contrast, that were still being harmed in 2011. There were no differences between the group that were no longer negatively harmed and the group that continued to be harmed (i.e. reported being harmed in both 2008 and 2011). For instance, there was no statistically significant difference in the percentage of men in the discontinuation group (30.6%) and the percentage of men in the continuation group (27.7%), and the younger group was also no more likely than respondents aged 36 years or more to report discontinuation of harm. There was also no statistically significant difference in discontinuation of harm between the more and less disadvantaged groups and groups that drank in different ways. The odds of reporting discontinuation of harm were not significantly raised or lowered in association with any of the explanatory variables.

Table 3 describes the types of harms respondents experienced because of their IPs in 2008 and seeks to understand whether the group that no longer experienced harm in 2011 was more or less likely to report harm than the group that continued to report harm. In the interim between the two surveys, the circumstances of a number of respondents changed; in that 25 out of the 83 respondents nominated someone else (e.g. son, father, friends) as their most harmful drinker. As their IP was no longer the person who most negatively affected them, the specific harms asked about in Table 3 were asked about the other relationship that was negatively affecting them. This reduced the sample in Table 3 to 58 and limited the power we had to investigate statistical difference even further. Being emotionally hurt or neglected, having a social occasion negatively affected and being involved in a serious argument because of an IP's drinking (in varying rankings) were the three most common specified harms reported in both groups. In general, both groups reported experiencing similar harms. There was no evidence from the bivariate logistic regression that more or less likely experience of any of the specific harms was associated with greater odds of continuation (or discontinuation) of harm from an IP's heavy drinking. Although the odds of continued harm were increased if the respondent had been in a serious argument (odds ratio [OR] = 1.6), been pressured or forced into sex (OR = 1.4), been emotionally hurt (OR = 1.8), or financially disadvantaged (OR = 2.1) or reported that the IP had failed in their role (OR = 1.5) because of their drinking, none of these results were statistically significant. In the other direction, although these results were also not statistically significant, for respondents who had been physically hurt (OR = 0.4), the odds that these respondents had continued to experience harm were decreased, as were respondents who reported that their IP's heavy drinking meant that they could not bring friends home (OR = 0.6). There were increased odds that respondents who experienced more types of harm were 1.5 times more likely to have continued to be harmed but there was no evidence of statistical difference between the groups.

## Discussion

While a substantial minority of respondents report having an IP who is a heavy drinker, only some of these report being negatively affected by the IP's drinking in the past year. Even so, that an estimated 6.7% of the Australian population report negative effects is of concern. Almost half of the respondents who have an IP who drinks heavily report being negatively affected by their drinking, with this figure higher in spousal and boyfriend/girlfriend relationships than in ex‐partner relationships.

While these findings suggest that the majority of the population did not experience harm from an IP because of their drinking in either of the 2 years asked about, harm from an IP's drinking does constitute a persistent problem for a notable proportion (3%) of the population. There are several possible ways of interpreting why things have not changed for this group. Presumably, neither the respondent nor the partner left the relationship in the interim, although it is possible that a few respondents may have left one relationship and taken on a new partner whose drinking is also harmful for them. Any efforts from either side of the relationship to make changes so that the harm ceases seem to have been unsuccessful.

Women reported negative effects from their IP's drinking more often than men at baseline. However, women were not (statistically) significantly more likely than men to report continuation of harm in the 2011 assessment period. Younger respondents were also no more likely than older participants to report continuation of being negatively affected by the harmful drinking of an IP. There was also no significant evidence that the respondent drinking five or more drinks at least monthly was associated with more or less reported harm from an IP's drinking despite the fact that concordant drinking between IPs has been previously shown to be linked to greater relationship satisfaction 23. However, these findings of no effect need to be treated with caution as the numbers in the study were small, and these results need to be re‐tested in studies with larger sample sizes.

A range of harms were commonly reported by those adversely affected by the drinking of IPs in 2008, but our study did not find statistically significant differences in reported discontinuation of harm by presence of particular types or numbers of types of problems. Thus, these findings are not very helpful in identifying whether some groups of respondents are more or less likely to reduce, tolerate, leave or ‘escape’ these harmful situations caused by their IPs. With larger sample sizes, studies of this type may be able to illuminate predictors of these changes. Using smaller samples, in depth qualitative studies would detail how IPs effectively manage or reduce the harms they experience, including whether they have done so by removing themselves from the situation or by asking their IPs to leave. Qualitative studies would also provide information on the types of services that respondents think may assist them either to stay and enable them to protect themselves and other family members or, alternatively, help them to separate or minimise contact with people who are harming themselves and their families.

### Limitations

For a small number of the items that were less specific (e.g. were you emotionally hurt because of your partner's drinking?), variability and subjectivity in individual responses—based upon alcohol expectancy, personal beliefs about alcohol use, self‐use, etc.—may have been introduced, assessing emotional states rather than the effects of alcohol in particular situations. However, the survey was designed to gain insight into the respondent's attribution of the event to alcohol. Alternative framings of the question, such as ‘did this occur while you were drinking’ are likely to give rise to higher counts of incidents that occurred while the respondent was drinking but not because of the drinking. For instance, in the Personal Safety Survey conducted in Australia in 2006, a smaller percentage of cases described attribution to alcohol than were described as occurring when the person held responsible was intoxicated 10.

While the data used in this study provide a unique perspective on the harms that people experience that are attributable to the drinking of others, the complex nature of social and familial networks dictates that there are limitations to what we can surmise from survey data. More detailed information about the respondent's relationship/s with intimate partners (e.g. when they started and finished and why) would enhance future surveys. In the case of this study, we know that our sample of interest experienced harm from an IP in 2008 that was attributed by the respondent to their partner's drinking and that some of these people reported the same thing in 2011. We do not know if this was because of the same IP, nor can we assume that the respondent left the drinker or anything else about their circumstances, instead we can just report on what it is we know; the correlates of this harm. There is also the possibility that the harms had escalated to the point that the respondent no longer felt safe enough to disclose the abuse. This scenario may have contributed to loss of sample, as suggested by Manton and Maclean 24 or to reporting of diminished harm despite its existence. Furthermore, as noted in the method section, the 2011 sample was not representative of the general population; therefore, it was only used to group the 2008 data, that is, all correlates reported on were derived from the first survey. In addition, the 2008 response rate, while the usual in an Australian context 25, is low by international standards, and this should be taken into account when interpreting results. Finally, the sub‐sample examined (*n* = 83) was small but was used to explore an important outcome.

## Conclusions

These findings underline the prevalence and nature of harms that occur in IP relationships, particularly to women, when the IP drinks heavily. These findings inform policy‐makers and advocates that almost half of the population group who experience harms from their IPs' drinking, continue to experience such harms over time. These harms include emotional harms, serious arguments, physical harm and inadequate role performance. No correlates predicted the discontinuation of harm but larger quantitative and additional qualitative studies are required to inform governments and non‐government agencies about who to focus on and how to act to reduce the harms experienced because of the heavy drinking of IPs.
